# An automated machine learning approach to predict brain age from cortical anatomical measures

**DOI:** 10.1002/hbm.25028

**Published:** 2020-05-16

**Authors:** Jessica Dafflon, Walter H. L. Pinaya, Federico Turkheimer, James H. Cole, Robert Leech, Mathew A. Harris, Simon R. Cox, Heather C. Whalley, Andrew M. McIntosh, Peter J. Hellyer

**Affiliations:** ^1^ Department of Neuroimaging Institute of Psychiatry, Psychology and Neuroscience, King's College London London UK; ^2^ Department of Psychosis Studies Institute of Psychiatry, Psychology and Neuroscience, King's College London London UK; ^3^ Center of Mathematics, Computation and Cognition Universidade Federal do ABC Santo André Brazil; ^4^ Division of Psychiatry University of Edinburgh Edinburgh UK; ^5^ Lothian Birth Cohorts group, Department of Psychology University of Edinburgh Edinburgh UK; ^6^ Scottish Imaging Network, A Platform for Scientific Excellence (SINAPSE) Collaboration Edinburgh UK

**Keywords:** age prediction, automated machine learning, cortical features, neuroimaging, predictive modelling, structural imaging

## Abstract

The use of machine learning (ML) algorithms has significantly increased in neuroscience. However, from the vast extent of possible ML algorithms, which one is the optimal model to predict the target variable? What are the hyperparameters for such a model? Given the plethora of possible answers to these questions, in the last years, automated ML (autoML) has been gaining attention. Here, we apply an autoML library called Tree‐based Pipeline Optimisation Tool (TPOT) which uses a tree‐based representation of ML pipelines and conducts a genetic programming‐based approach to find the model and its hyperparameters that more closely predicts the subject's true age. To explore autoML and evaluate its efficacy within neuroimaging data sets, we chose a problem that has been the focus of previous extensive study: brain age prediction. Without any prior knowledge, TPOT was able to scan through the model space and create pipelines that outperformed the state‐of‐the‐art accuracy for Freesurfer‐based models using only thickness and volume information for anatomical structure. In particular, we compared the performance of TPOT (mean absolute error [MAE]: 4.612 ± .124 years) and a relevance vector regression (MAE 5.474 ± .140 years). TPOT also suggested interesting combinations of models that do not match the current most used models for brain prediction but generalise well to unseen data. AutoML showed promising results as a data‐driven approach to find optimal models for neuroimaging applications.

## 1 INTRODUCTION

The last few decades have seen significant progress in neuroimaging methodologies and techniques focused on identifying structural and functional features of the brain associated with the behaviour. However, only a few of the newly developed methods have been transferred to the clinics. One of the principal reasons for this gap is that, so far most of the findings in the neuroscience field have been obtained by assessing differences at the group level (e.g., analysed the difference in brain activation in healthy controls compared to schizophrenia patients); however, decisions in the clinics need to be done at the individual level. Machine learning (ML) has been recently gaining attention as it promises to bridge the gap between group‐level analysis and individual inference. In fact, with the advance of ML algorithms and their increased application in neuroimaging, the field is rapidly becoming more focused on developing clinically relevant biomarkers, as well as, exploring relationships between individual differences and behaviour (Bzdok & Ioannidis, 2019; Pereira, Mitchell, & Botvinick, 2009; Shen et al., 2017; Yarkoni & Westfall, 2017).

One of the most promising uses of the brain age prediction is its relevance and use as a biomarker to assess the risk of an individual to develop cognitive decline and propensity to neurodegenerative diseases (Cole, Franke, & Cherbuin, 2019; Franke & Gaser, 2019). The main idea is that brains that are predicted to be older than their chronological age have aberrant age changes accumulation and that this accumulation might be a marker for disease and its progression. Supporting this idea, the brain‐age gap (i.e., the difference between brain‐age predicted and chronological age) has been shown to be higher in mild cognitive impairment who progress to Alzheimer's disease (Franke & Gaser, 2012), traumatic brain injury (Cole et al., 2018) and schizophrenia (Koutsouleris et al., 2014) patients when compared to controls.

Predictive modelling approaches, which consist of using ML algorithms to learn patterns from features in a data set and to build an accurate model to predict an independent variable of interest in unseen data, are gaining increasing attention in the neuroscience field. However, choosing a model which is unsuitable for the statistical distribution the underlying data leads to significant problems with overestimation of the model and loss of generalisation. Second, the sheer *mass* of learning approaches that are available with a vast array of different properties provides a bewildering set of choices for the practitioner; each with advantages and disadvantages both in terms of generalisation and computational complexity. This issue results in the occurrence of both Type I and II errors, simply as a result of picking an inappropriate analysis technique for the underlying data. This is particularly problematic as new fields adopt ML approaches, and the choice of the methodology is often based on applications in other fields where data may have quite different statistical properties—or indeed simply be the product of whichever technique is currently in the zeitgeist. A similar problem has been described and extensively studied in motion correction in resting state fMRI. Power et al. (2014) and Power, Schlaggar, and Petersen (2015) analysed the effect of different commonly used motion correction steps and how they change the statistical structure of the data set. These transformations not only have a significant effect on voxel‐level inference (Power et al., 2014, 2015) but also on cluster correction (Eklund, Nichols, & Knutsson, 2016). Interestingly, Eklund et al. (2016) showed that by violating the statistical properties of the data, the analysed parametric methods resulted in a very high degree (up to 70% instead of the usually assumed 5%) of false positives.

The *no free lunch principle* (Wolpert & Macready, 1997) applied to ML suggests that there are no single estimator and parameter combinations that will always perform well on every data set. The selection of preprocessing steps, the choice of the algorithm, the selection of features and the model's hyperparameters are crucial and will vary with the task and data. Hence, the optimal application of ML technology requires the answer to at least three questions: What are the necessary preprocessing steps that should be performed to prepare the data? Is there a way of reducing the feature space to only the relevant features? Among the many available ML algorithms, which one is the most appropriate for the data under analysis? That these choices are often arbitrary and defined only on *prior* wisdom is a challenge for neuroimaging which continues to face a significant replication crisis (Open Science Collaboration, 2015).

ML algorithms vary greatly in their properties, complexity and the assumptions they make about the data they are applied to. They can be linear, non‐linear and optimise different functions to predict continuous (regression) or categorical (classification) variables. Moreover, the performance of all ML algorithms depends on the fine‐tuning of its hyperparameters (Jordan & Mitchell, 2015). In addition, feature extraction and feature selection methods are often used in series to reduce or enhance data complexity during the preprocessing stages of analysis. The consequence is that there are potentially infinite combinations of approaches that can be taken to identify relationships out of data. To cut through this complexity requires the development of tools that can automatically select the appropriate (combination of) preprocessing and ML techniques to apply to a data set to highlight relationships that are both generalisable and computationally efficient.

**FIGURE 1:**
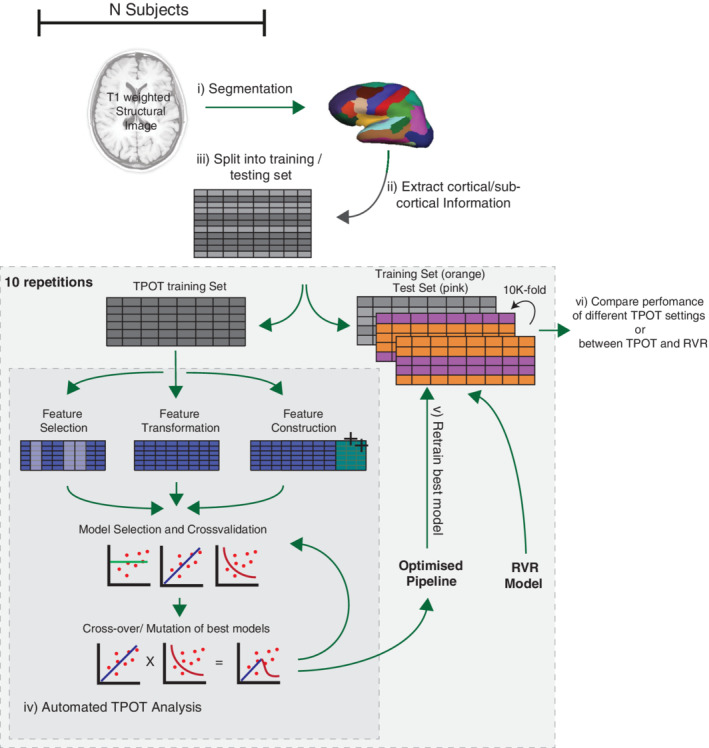
Overview of experimental design. The subject's structural MRI is used to create a parcellation of cortical and subcortical regions. The data set was split into two independent sets: TPOT training set and evaluation set. The TPOT training set was passed to TPOT, which depending on the specified configuration performed feature selection, feature transformation, feature generation or a combination of those and evaluated the model's performance. For each generation, a 10‐fold cross‐validation was performed and the best models for that specific generation were identified, crossed‐over/mutated and passed to the next generation. At the last generation, the pipeline with the lowest mean absolute error (MAE) was identified and returned by TPOT. We then retrained the optimised pipeline on the independent evaluation set and tested its performance using a 10‐fold cross‐validation. Finally, we compared the MAEs between different TPOT configurations and between TPOT and RVR

In recent years, automated ML (autoML) has been gaining attention. The aim of autoML is to take advantage of complexity in the underlying data set to help guide and identify the most appropriate model (and their associated hyperparameters), optimising performance, whilst simultaneously attempting to maximise the reliability of resulting predictions. In this context, many different autoML libraries have been developed. Auto‐WEKA (Thornton, Hutter, Hoos, & Leyton‐Brown, 2013), Auto‐Sklearn (Feurer et al., 2015) and Tree‐based Pipeline Optimisation Tool (TPOT; Olson, Bartley, Urbanowicz, & Moore, 2016) are just a few examples. Although the first two implement a hierarchical Bayesian method, the latter uses a tree‐based genetic programming algorithm. Due to its user‐friendly interface and the pipeline flexibility offered by the optimisation of a tree‐based approach (Hutter, Kotthoff, & Vanschoren, 2019), we have chosen to evaluate TPOT's performance on this problem. The main idea behind the tree‐based genetic programming is to explore different pipelines (i.e., combination of different operators that perform features selection, feature generation and model analysis) for solving a classification or regression problem. This is done through a multi‐generation approach, starting from a collection of *random* models. Based on the performance and reliability of predictions at each generation those with the highest performance will be *bred* (i.e., combined or crossed‐over), whilst random *mutations* of these models are also introduced. Therefore combinations of models that maximise both performance and have lower complexity survive and the “best” candidate pipeline yielded by TPOT will consist of a combination of models and preprocessing methods that are best suited to the relationship being probed. Figure 1 presents a high‐level schematics of our approach.

In this article, we explore the application of TPOT as an autoML approach to structural neuroimaging data. As a test case, we evaluated its efficacy to predict chronological age using structural brain data. Ageing is one factor inducing major variability in brain structure. Grey matter atrophy, increase in the ventricle sizes and cortical thinning are a few examples of structures that alter whilst we age (Cole & Franke, 2017; Hogstrom, Westlye, Walhovd, & Fjell, 2013). As age‐related changes can be detected with structural MRI, different ML models have been trained to learn the relationship between age and brain structure (Aycheh et al., 2018; Becker, Klein, Wachinger, Initiative, et al., 2018; Cole, Leech, Sharp, & Initiative, 2015; Franke et al., 2010; Liem et al., 2017; Madan & Kensinger, 2018; Valizadeh, Hänggi, Mérillat, & Jäncke, 2017). The main idea behind brain age studies is to find discrepancies between the predicted and chronological age, which might be used as biomarkers (Cole & Franke, 2017). As brain‐age prediction has been extensively studied and its accuracy can be evaluated against the reported model accuracies the existing brain‐age corpus (Aycheh et al., 2018; Cole et al., 2017; Franke et al., 2010; Valizadeh et al., 2017), we used this problem to test the settings, validity and limitations of autoML for imaging applications in using a regression approach. In this study, we demonstrate that: (a) the model's performance is highly dependent on the initial model population defined by the initial model pool passed as a configuration and the population size; (b) there is no single analysis model that predicts age with the highest performance from the underlying structural imaging data and (c) models suggested by TPOT outperforms relevance vector regressor (RVR), a state‐of‐the‐art model used to predict brain age. Therefore, TPOT can be used as a data‐driven approach to learn patterns in the data, to automatically select the best hyperparameters and models in a researcher unbiased fashion to avoid common pitfalls from ML algorithms such as overfitting.

## 2 MATERIALS AND METHODS

### 2.1 Subjects and data sets

In this analysis, T1‐weighted MRI scans from *N* = 10,307 healthy subjects (age range 18–89 years, mean age = 59.40) were obtained from 13 publicly available data sets where each data set used one or more scanners to acquire the data. A summary of the demographics and imaging information can be found in Table 1 (for more details about the BANC data set, see Cole et al., 2017) and for the UK Biobank (Alfaro‐Almagro et al., 2018; Sudlow et al., 2015; https://biobank.ctsu.ox.ac.uk/crystal/crystal/docs/brain_mri.pdf). From the original *n* = 2,001 subjects present on the BANC data set, we only used 1,227 subjects and excluded all subjects from the WUSL Cohort. The WUSL Cohort, in particular Cohort 3 that contained 26 adult subjects (Power, Barnes, Snyder, Schlaggar, & Petersen, 2012), was excluded after performing Freesurfer quality control checks. The exclusions were made based on poor quality automated labelling, whereby non‐brain tissue was included in the grey matter segmentation. This was likely driven by noise or artefacts in the original images.

**TABLE 1:** Overview of the demographics and imaging parameters for the different datasets

Cohort	N	Age mean (*SD*)	Age range	Sex male/female	Repository details	Scanner (field strength)	Scan	Voxel dimensions
ABIDE (Autism Brain Imaging Data Exchange)	147	24.43 (4.89)	18–40	130/17	INDI	Various (all 3T)	MPRAGE	Various
Beijing Normal University	151	21.36 (1.95)	18–28	63/88	INDI	Siemens (3T)	MPRAGE	1.33 × 1.0 × 1.0
Berlin School of Brain and Mind	49	30.99 (7.08)	20–60	24/25	INDI	Siemens Tim Trio (3T)	MPRAGE	1.0 × 1.0 × 1.0
CADDementia	12	62.33 (6.26)	58–79	9/3	http://caddementia.grand‐challenge.org	GE Signa (3T)	3D IRFSPGR	0.9 × 0.9 × 1.0
Cleveland Clinic	31	43.55 (11.14)	24–60	11/20	INDI	Siemens Tim Trio (3T)	MPRAGE	2.0 × 1.0 × 1.2
ICBM (International Consortium for Brain Mapping)	42	27.71 (5.75)	24–60	14/28	LONI IDA	Siemens Magnetom (1.5T)	MPRAGE	1.0 × 1.0 × 1.0
IXI (Information eXtraction from Images)	394	46.21 (16.11)	20–86	159/235	http://biomedic.doc.ic.ac.uk/brain‐development	Philips Intera (3T); Philips Gyroscan Intera (1.5T); GE Signa (1.5T)	T1‐FFE; MPRAGE	0.9375 × 0.93751 × 1.2
MCIC (MIND Clinical Imaging Consortium)	92	32.33 (11.92)	18–60	63/29	COINS	Siemens Sonata/Trio (1.5/3T); GE Signa (1.5T)	MPRAGE; SPGR	0.625 × 0.625 × 1.5
MIRIAD (Minimal Interval Resonance Imaging in Alzheimer's Disease)	23	69.66 (7.18)	58–85	12/11	https://www.ucl.ac.uk/drc/research/miriad‐scan‐database	GE Signa (1.5T)	3D IRFSPGR	0.9375 × 0.93751 × 1.5
NEO2012 (Adelstein, 2011)	39	29.59 (8.38)	20–49	18/21	INDI	Siemens Allegra (3T)	MPRAGE	1.0 × 1.0 × 1.0
Nathan Kline Institute (NKI)/Rockland	151	41.92 (18.24)	18–85	94/57	INDI	Siemens Tim Trio (3T)	MPRAGE	1.0 × 1.0 × 1.0
OASIS (Open Access Series of Imaging Studies)	61	42.82 (20.42)	18–89	20/41	http://www.oasis‐brains.org/	Siemens Vision (1.5T)^a^	MPRAGE	1.0 × 1.0 × 1.25
TRAIN‐39	35	22.77 (2.52)	18–28	10/25	INDI	Siemens Allegra (3T)	MPRAGE	1.33 × 1.33 × 1.3
UK BIOBANK	9080	62.45 (7.48)	45–79	4334/4746	https://biobank.ctsu.ox.ac.uk/crystal/crystal/docs/brain_mri.pdf	Siemens Skyra (3T)	MPRAGE	1.0 × 1.0 × 1.0
**Training set total**	**10307**	**59.40 (12.33)**	**18–89**	**4961/5346**	**‐**	**‐**	**‐**	**‐**

*Note*. ABIDE consortiums comprising data from various sites with different scanners/parameters.

Abbreviations: COINS, Collaborative Informatics and Neuroimaging Suite (http://coins.mrn.org); INDI, International Neuroimaging Data‐sharing Initiative (http://fcon_1000.projects.nitrc.org); LONI, Laboratory of Neuro Imaging Image & Data Archive (https://ida.loni.usc.edu).

a OASIS scans were acquired four times and then averaged to increase signal‐to‐noise ratio.

### 2.2 MRI preprocessing

Using the recon‐all pipeline in Freesurfer version v6.0 (Dale, Fischl, & Sereno, 1999), individual T1‐weighted MRI images were preprocessed and parcelled into 116 thickness and volume information for anatomical structures (for the full list of features, see Table S2), according to the Desikan‐Killiany atlas and ASEG Freesurfer atlas (Desikan et al., 2006). From these segmented regions, we extracted the cortical thickness and volume to be the input data for our further analysis.

### 2.3 TPOT automated analysis

TPOT (Olson, Bartley, et al., 2016; Olson, Urbanowicz, et al., 2016) uses genetic programming to search through different operators (i.e., preprocessing approaches, ML models, and their associated hyperparameters) to iteratively evolve the most suitable pipeline with high accuracy. It does so by (a) generating a pool of random analysis models sampled from a dictionary of preprocessing approaches and analysis models (see Table S1 for a list of the models used); (b) evaluating these models using 10‐fold cross‐validation, to identify the most accurate pipeline with the lowest amount of operators; (c) breeding the top 20 selected pipelines and applying local perturbations (e.g., mutation and crossover) and (d) re‐evaluating the pipeline in the next generation. This process is repeated for a specified number of generations before settling on a final optimal pipeline that has high accuracy and low complexity (i.e., lowest number of pipeline operators). To make sure that the operators are combined in a flexible way, TPOT uses a tree‐based approach. That means that every pipeline is represented as a tree where the nodes represented by the different operators. Every tree‐based pipeline starts with one or more copies of the data set and every time the data are passed through a node, the resulting prediction is saved as a new feature. In particular, TPOT uses a genetic programming algorithm as implemented in the Python package DEAP (Fortin, Rainville, Gardner, Parizeau, & Gagné, 2012; for a more detailed description of the TPOT implementation, see Olson, Bartley, et al., 2016). The models used for TPOT included a combination of linear (interpretable) and non‐linear models (non‐interpretable). A list of all models for feature selection, feature generation and regression used for the TPOT analysis and their scikit‐learn implementation can be found in Table S1.

#### 2.3.1 Regression

##### TPOT hyperparameters exploration

We used TPOT to find the “best” pipeline to predict brain age, where the fitness of the pipeline is defined by a low mean absolute error (MAE) between the predicted and the subject's chronological age. To do this, we randomly selected 1,546 subjects from the data set (TPOT training set), and we applied TPOT on them for 10 generations to find the most fitted ML pipeline—the pipelines with the highest accuracy. The optimal pipeline suggested by TPOT was then used to train an independent (*n* = 8,761) data set and its performance was evaluated using a 10‐fold cross‐validation. Both RVR and the optimal model suggested by TPOT were trained using the same number of subjects. The TPOT analysis and the evaluation of the model in an independent training set were repeated 10 times. As a result, we obtained 100 performance scores for each configuration that were used to evaluate the impact of manipulating (a) the types of model preprocessing, (b) number of models tested on the first generation and (c) mutation and crossover rate.

### 2.4 Relevance vector regression

RVR was first introduced by (Tipping, Solla, & Leen, 2000) and uses a general linear model based on Bayesian inference and therefore, in contrast to most commonly used models, it returns probabilistic predictions instead of deterministic predictions. In addition, for the usage of this algorithm, the only hyperparameter that needs to be defined by the researcher is what type of kernel to use. In this experiment, we have used a linear kernel. All the other parameters are estimated by the model during the learning procedure. This avoids the need of cross‐validation that can increase the computational expenses for training the model. Another advantage is that RVR normally leads to sparser models resulting in a good generalisation error whilst having a faster prediction performance on the test data. However, the algorithm is more prone to local minima as its optimisation is non‐convex (Tipping et al., 2000).

### 2.5 Comparison between TPOT and RVR

We also performed a 10 times repetition with 10‐fold cross‐validation (as described above) to assess the difference in performance between the “best” pipelines yielded by TPOT and the RVR, a standard model used in brain‐age prediction (Franke et al., 2010; Kondo et al., 2015; Madan & Kensinger, 2018; Wang et al., 2014). In addition, to check if the underlying age distribution would have an effect on the models yielded by TPOT, we repeated the analysis using 784 subjects whose age was uniformly distributed between 18 and 77 years old. In this case, we used *n* = 117 subjects to train TPOT and obtain the best pipeline. The remaining subjects (*n* = 667) were used to train the best pipeline using a 10‐fold cross‐validation. Similarly to the other analyses, this evaluation process was also repeated 10 times resulting in 100 MAE values for each condition.

Although a Student's *t* test is often used to check the difference in performance between two models, Student's test assumes that samples are independent, an assumption that is violated when performing a k‐fold cross‐validation. As part of the k‐fold cross‐validation procedure, one subject will be used in the training set k−1 times. Therefore, the estimated scores will be dependent on each other, and there is a higher risk of Type I error. In fact, Nadeau and Bengio (2003) observed that he increase of Type I error is given by an underestimation of the variance as the samples are not independent. The corrected *t*test is defined as following Nadeau and Bengio (2003):

(1) $$t=\frac{\frac{1}{n}\sum_{j=1}^{n}a_{j}-b_{j}}{\sqrt{\left(\frac{1}{n}+\frac{n_{2}}{n_{1}}\right){\hat{\sigma }}^{2}}}$$

where *a*_*j*_ and *b*_*j*_ are the accuracy of the two algorithms being compared, *n*_1_ are the instances used for training and *n*_2_ the instances for testing. The major difference is that the $\frac{1}{n}$ factor in the denominator has been replaced by the factor $\frac{1}{n}+\frac{n_{2}}{n_{1}}$. For this reason, we used a corrected version of the *t* test that accounts for this dependency (Nadeau & Bengio, 2003) when comparing the performance of TPOT and RVR and the Friedman test when comparing different hyperparameters from TPOT (Demšar, 2006).

## 3 RESULTS

**FIGURE 2:**
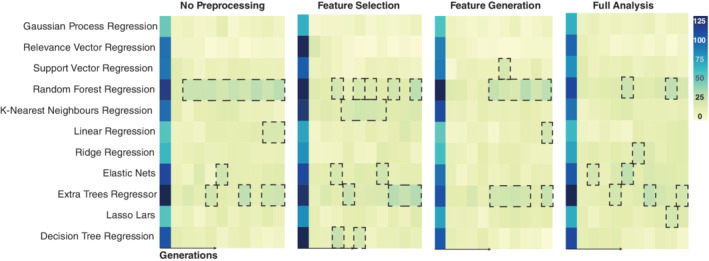
Overview of the models count for each generation from one repetition for the different configurations experiments. Models with a darker colour were more popular then models with lighter colour. Across the four experiments, random forest, K‐nearest neighbours, linear regression and extra trees regressors are the models with the highest count per generation. To make sure that all models were represented, we had 1,000 models in the first generation and 100 models were passed on for the following generations

We firstly investigated which models survived through the different generations. Figure 2 shows the counts of the different models in one of the repetitions. Random forests and extra‐trees regressors are the most popular models followed by Elastic Nets. Decision trees and k‐nearest neighbours also have a high popularity for the feature selection configuration.

### 3.1 TPOT parameter exploration

**FIGURE 3:**
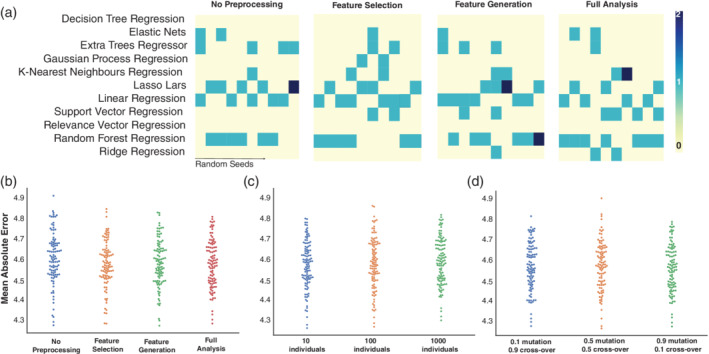
Overview of the ensembles for the different analysis configurations at each repetition and their performance. (a) Schematic overview of the models composing the ‘best’ ensembles yielded by TPOT at each repetition. A darker colour represents models with higher counts. Random forest regression, extra trees regressors, lasso lars and linear regression were the most frequently represented. Despite the different models combinations among the different preprocessing analysis (b), initial population size (c) and mutation/cross‐over rate (d), there was no difference in the yielded performance

We then explored if the changes in the TPOT configuration are associated with a different performance (Figure 3b). We observed that independent of the preprocessing, the analysis choses the performance varied between 4.3 and 4.9 years. If there was a single best model to predict brain age, we would expect this model to always be identified by the automated pipeline and included in the ensembles. However, what we observed was that for every repetition, TPOT found a different pipeline which was considered to be the most accurate and none of the models were consistently identified throughout the repetitions. Nevertheless, some of the models such as linear regression, lasso lars and random forest regression seem to be popular choices. (Figure 3a). Similarly, we analysed the change in performance when varying the initial population of pipelines (Figure 3c). If a model was not selected on the initial population, it will never be present in future generations; therefore, we expected that a larger initial population would lead to a more diverse pool and be associated with higher performances. We also explored the effect of mutation and crossover rate on the performance of the derived pipelines. For a combination of high (0.9), low (0.1), mid‐ranges (0.5) mutation and cross‐over rates, see Figure 3d. Another key factor suggesting that there is not a best model to predict brain age is that for all tested configurations, the performance of the best models yielded by TPOT oscillated between 4.3 and 4.9 years (Figure 3b–d).

These suggest that there is not one single model that best describes the data set but a combination of many models leads to a higher performance, and independent of the of the underlying data structure, TPOT was able to a pipeline that yielded high performance.

### 3.2 Comparison between TPOT and RVR

**FIGURE 4:**
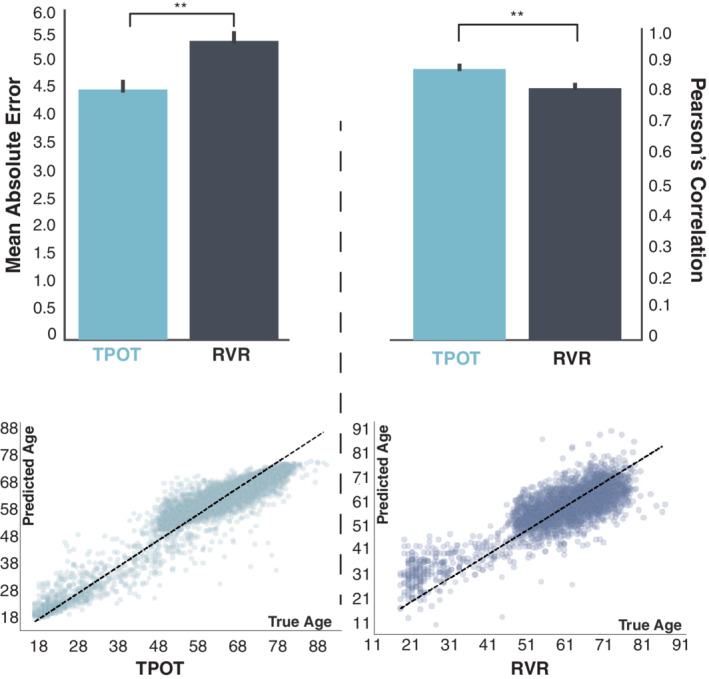
Comparison of model's performance between TPOT and RVR. We compared the MAE (top panel left) and Pearson's correlation (top panel right) between true and predicted age of the optimised model suggested by TPOT with and RVR on the test set. The lower panels show the predicted versus the true age for one of the optimal pipelines suggested by TPOT (left) and RVR (right). Note that although both models use the same subject to make prediction, the scales of the TPOT and RVR predictions are different, and the RVR model predicts young subject to be younger and old as older. Asterisks show differences that are statistically significant at *p* < .01 (*t*‐test corrected); error bars indicate ±1*SD*

To assess the efficacy of the TPOT approach applied to neuroimaging data, we compared the performance of the TPOT's pipelines using the full analysis configuration with RVR. When using the entire data set, TPOT had a lower MAE and higher Pearson's correlation between true and predicted age (Figure 4). However, when we applied TPOT to a uniformly distributed data set, there was no significant difference between the models yielded by TPOT and RVR (Table 3). As the performance of the algorithms strongly depends on the number of samples used to train it, it is hard to disentangle if the observed decrease in accuracy was due to the enforced uniform distribution of the data or because of its reduced sample size. Nevertheless, the models suggested by TPOT using both data sets with the different age distribution were similar. Both Figure S1 and Figure 2, which depict the count of the most common models in the uniform and unchanged distribution respectively, illustrate that the most commonly selected models included random forest regressions, elastic nets and extra‐trees regressors. Together, these results suggest that the models suggested by TPOT for brain age prediction were invariant to the data sampling bias for the current data set.

To facilitate the comparison between the models, we also provide the computational time for the different methods in Table 2. The analysis was performed using an Intel Xeon CPU E5‐2640 v4 (2.40 GHz). The TPOT training, that is the process in which TPOT is searching for the optimised pipeline, is by far the most consuming step taking approximately 6 hr to find the optimal pipeline. However, it should be noted that these values represent the time needed to run 10 generations of the genetic algorithm with five cross‐fold validation, and we did not use any parallelisation strategies. Retraining the best TPOT, pipeline in an independent data set then takes about 5 min and the inference another 0.174 s. On the other hand, the RVR models takes about 8 min to train and 0.001 s to perform the inference on an external data set.

**TABLE 2:** Comparison between TPOT and RVR time‐complexity

	RVR	TPOT
TPOT training	‐	About 6 hr (6.61 ± 0.39 hr)
Training	About 8 min (519.37 ± 2.62 s)	About 5 min (276.02 ± 2.51 s)
Inference	0.001 ± 3.85*e* ^−05^s	0.174 ± 0.06 s

## 4 DISCUSSION

The successful choice of an ML pipeline to predict variables of interest (such as age) from neuroimaging data is driven by the statistical characteristics and distribution of the data set under analysis. In most cases, the choice of ML model applied in multivariate analysis of neuroimaging data is rather arbitrary—based on prior models that “have worked,” or by selecting whichever model is most novel in the eyes of the analysis community. To explore an alternative approach to model selection for a relatively simple problem, in this work, we investigated the application of an automated analysis technique: TPOT. The TPOT approach is a data‐driven methodology which is agnostic to statistical model *and* prepossessing of the data set—aiming to find the best pipeline available to fit the statistical properties of the underlying data set, whilst simultaneously controlling for overfit and reliability. We showed that: (a) the performance of the models suggested by TPOT is highly dependent on the specified model pool (i.e., algorithms and hyperparameters) that TPOT has available to use. However, feature selection, feature generation, initial population size the mutation rate and cross‐values rate do not have a substantial effect on the TPOT's performance. (b) There is not one single ML algorithm that performs the best, but good performance is achieved by a combination of models. (c) The pipelines suggested by TPOT performed significantly better than commonly used methods when performing a brain age regression from brain MRI scans.

Different neuroimaging methodologies functional MRI (Dosenbach et al., 2010), diffusion MRI (Richard et al., 2018) and structural MRI (Cole et al., 2017; Corps & Rekik, 2019; Franke et al., 2010) have been used to study the association between the changes in brain structure and ageing. Commonly used algorithms to predict brain age include a combination of linear and non‐linear ML algorithms such as: multiple linear regression (Valizadeh et al., 2017), Gaussian process regressors (Becker et al., 2018; Cole et al., 2015), K‐nearest neighbours (Valizadeh et al., 2017), RVR (Franke et al., 2010; Valizadeh et al., 2017; Wang et al., 2014), random forests (Valizadeh et al., 2017), connectome‐based predictive modelling (Corps and Rekik (2019) and neural networks (Cole et al., 2017; Valizadeh et al., 2017). In this study, we used an autoML approach that searched for the most accurate pipeline over a pool of the commonly used algorithms and compared its performance to RVR. We observed that the variance in the predicted accuracy is very low on the test data set for the pipelines suggested by TPOT but also for the RVR model. This suggests that the models are not fitting to noise but are finding interesting patterns in the data. Nevertheless, it is interesting to note that for every analysis's repetition, a different pipeline was yielded by TPOT which had the lowest MAE (i.e., “best” pipeline; Figure 3a). This is likely because there exists no single model that always performs better for this type of regression problem.

Similarly, when analysing age prediction using voxel‐wise data Varikuti et al. (2018) showed that the pattern of “important” voxels is different across different training sets. Given the strength of the association between brain structure and age, and high levels of correlation between different brain regions, it seems that multiple different approaches can achieve high levels of prediction accuracy. As it seems that different weighting on the brain could reach a similar level of performance, interpretation of model weights or coefficients should be done with caution. Inference on which brain regions are most associated with ageing is better conducted using a longitudinal within‐subjects study design, rather than a multivariate predictive model such as those used in TPOT. Our results also highlight that all models yielded a similar MAE and were composed by a combination of linear and non‐linear models (random forest regression, extra tree regression, K‐nearest neighbours and ridge or lasso regression; Figures 2 and 3). In accordance with our results, Valizadeh et al. (2017) also reported similar brain‐age prediction accuracy when comparing random forest and multiple linear regression. One of the main advantages of random forests is that it can deal with correlated predictors, whilst in a linear regression, correlated predictors might bias the results. Therefore, by combining both algorithms in an ensemble, TPOT combines the strengths of both algorithms. Random forests have also been used by Liem et al. (2017) to combine multi‐modal brain imaging data and generate brain‐age prediction. In particular, Liem et al. (2017) used a linear support vector regression to predict age and stacked these models with random forests. This combined approach was able to improve brain‐age prediction. Our interpretation of these observations is that the use of random forests and the hyperparameters found by TPOT “better fit” the non‐trival non‐linearities present in the data set, transforming them within an *n*‐dimensional manifold which can then be fed trivially into a linear classifier. A similar observation has been described by Aycheh et al. (2018), where a combination of sparse group lasso and Gaussian process regression was used to predict brain age. On the other hand, whilst stable, and able to generalise, this non‐linear transformation and combinations of different models into a pipeline makes interpretation of important features within the data set impossible.

We also noted that when using a subsample of the data set that has a uniform distribution, similar models were used by TPOT to build ensembles, nevertheless the difference in performance between TPOT and RVR was not significant (Table 3). We hypothesise that by using a uniform distribution, we make the problem of age regression easier and therefore obtained similar performance between the TPOT and RVR approach, or that the reduced sample used to pre‐train TPOT was not sufficient to obtain an accurate fit. It would be interesting for future research to explore these hypotheses further.

**TABLE 3:** Comparison between TPOT and RVR. Although TPOT has a significant higher accuracy and Pearson's correlation when using the original data distribution, when using the uniformly distributed data set both models had a similar performance (the values represent ±*SD*)

	MAE	*p* Value	*t*	Pearson's correlation	*p* Value	*t*
TPOT	4.612 ± .124	<**.01**	−6.441	.874 ± .012	<**.01**	3.745
RVR	5.474 ± 0.140			.813 ± .0102		
TPOT (uniform distribution)	5.594 ± .0706	>.5	−0.616	.917 ± .027	>.5	0.007
RVR (uniform distribution)	5.975 ± .525			.919 ± .013		

*Note*. The bold values correspond to analysis with a significant *p*‐value (*p* < 0.05).

In the context of other literature, it is important to note that more accurate brain‐age prediction models do exist. As shown by Cole et al. (2017), convolutional neural networks can predict brain age with an MAE of 4.16 years using a similar age range (18–90 years, mean age = 36.95). In addition, Peng, Gong, Beckmann, Vedaldi, and Smith (2019) also developed a simple fully convolutional network that could predict age in the UK Biobank data (44–80 years, mean age 52.7 years) with MAE of 2.14 years. As developing neural networks require in‐depth knowledge of architecture engineering, it would be interesting to use autoML approaches to explore and select the most appropriate network architecture. In the specific case of deep‐neural network approaches to the brain age problem, whilst improvements can be made on the accuracy of the model, often this is at the cost of reliability. As TPOT can accommodate a wider set of models, it would be interesting to include neural networks on the model pool and compare its performance against the range of selected models or to use other autoML toolboxes like autokeras (Jin, Song, & Hu, 2019) or efficient neural architecture search via parameter sharing (Pham, Guan, Zoph, Le, & Dean, 2018). In a very interesting and innovative work, Xie and Yuille (2017) explored the possibility of constructing deep learning networks structures automatically using a genetic algorithm approach to explore a vast search space. Although their algorithm did not explore all possible network structures, their results showed good performance on traditional ML data sets and highlight the promising advances we will see in this field. Similar automated approaches will allow an extensive search of models and parameters and might also shed light into the question if deep learning is beneficial to neuroimaging analysis. Recently, Schulz et al. (2019) showed that linear, kernels and deep learning models show very similar performance in brain‐imaging data sets. Combining the potential power of deep learning with a model‐agnostic technique, such as employed by TPOT, offers a potentially interesting route for further research.

One of the main limitations of our study is feature interpretability. The pipelines built by TPOT are formed by concatenating different algorithms, and therefore by using TPOT it becomes very difficult to track the importance of the features of the algorithms. We consequently did not explore the relevance of the different features in this study. At the same time that the ensembled approach is one of the main limitations of our current analysis, it is also one of its biggest strengths. The combination of multiple models allows for the compensation of different weaknesses and strengths of the models, and therefore combining different models leads to an improvement on the pipeline performances.

For our analysis, we choose to use the RVR as a benchmark for the TPOT performance not only because this is the most commonly used model to predict brain age (Franke et al., 2010; Franke & Gaser, 2019), but also because the algorithm does not require any parameter optimisation (Tipping et al., 2000). Some recent studies carried out with large data sets showed that, independent of the model, the achieved performance to predict brain age is similar. For example, Han et al. (2019) showed that after a 10‐fold cross‐validation Gaussian process regression, ridge regression, generalised additive models and SVR all showed similar performances. Therefore, we did not benchmark the performance of all 11 models used on the TPOT model pool as it would be computational and resources costly and the results would not add significance to the article.

We would also like to point out that the problem of finding the best algorithm for a specific problem depends not only on the data set under analysis but also on the algorithm of choice. Regarding the impact of the data set (i.e., age range and distribution) on this article, we discuss how different data sets lead to different results. First, we used our approach and compared the entire data set to a uniform distribution. We observed that by changing the distribution of our data set, we obtained a worse performance (Table 3). In addition, when comparing the accuracy of different studies, it is important to take into account the age range of the analysed sample, as age prediction in a small range has less variability than in a large range. In fact, using a sample with subjects aged 45 to 91, Aycheh et al. (2018) obtained a MAE of 4.02 years. Although Valizadeh et al. (2017) had a similar age range as that described in our project, they do not report the MAE for the entire sample and use instead three age groups (8–18, 18–65 and 65–96 years) to test the accuracy of different models. In general, Valizadeh et al. (2017) reported lower accuracy for the older group with MAE ranging between 4.90 and 14.23 years, when using only the thickness information. On the other hand, Liem et al. (2017) using only the cortical thickness reported a MAE of 5.95 years (analysed age range 18–89 years, mean = 58.68). The second point to take into account when finding the *best* algorithm is the performance and tuning of the algorithm which will be specific to the training data set. As we know from the adaptive statistics literature (Turkheimer, Pettigrew, Sokoloff, & Schmidt, 1999), it may well be that certain algorithms will fit better certain data distribution; however in practice, one generally does not know the statistical distribution of the data hence adopting one model only is very likely to lead to a worse performance. The power of the currently used method relies on the fact that the researcher does not need to know the data set statistical distribution in order to find the most appropriate model. All is done automatically by TPOT.

In addition, with this article, we do not want to find the most accurate model to predict brain age. We want to test how well a completely automated pipeline can be in finding the most appropriate model for the data set under analysis and how well it performs compared to the most commonly used model. The main idea behind this is to extend the usage of ML to many researchers that are not familiar with the underlying statistical properties of different models and allow them to find good algorithms that generalise well.

## 5 CONCLUSION

Overall, our results show that the TPOT approach can be used as a data‐driven approach to find ML models that accurately predict brain age. The models yielded by TPOT were able to generalise to unseen data set and had a significantly better performance than RVR. This suggests that the autoML approach is able to adapt efficiently to the statistical distribution of the data. Although more accurate brain‐age prediction models have been reported (Cole et al., 2017), the approach in the present study uses a wide age range (18–89 years old), uses only cortical anatomical measures, but most of all, it does not make any assumptions about the underlying statistics of the data set and does not require any fine‐tuning of the model of choice. By extensively testing different models and its hyperparameters, TPOT will suggest the optimal model for the training data set. This approach removes possible introduced bias out of the loop and allows decisions about the model to be made in an automated, data‐driven and reliable way.

## CONFLICT OF INTEREST

The authors declared no potential conflict of interests.

## AUTHOR CONTRIBUTIONS

J.D., W.P., F.T., J.C., R.L., and P.H. designed the study. M.E., S.C., H.W., A.M., and J.C. preprocessed the data set. J.D. performed the experiments. J.D., W.P., and P.H. analysed the data. J.D., P.H., W.P., F.T., J.C., S.C., and H.W. wrote and edited the manuscript.

## Supporting information


**Appendix** **S1.** Supporting Information.

## Data Availability

All datasets used for this study are included in the manuscript/supplementary files.
