# Supplementary material for: An automated machine learning approach to predict brain age from cortical anatomical measures
Source: Hum Brain Mapp. 2020 May 16;41(13):3555–66. doi: 10.1002/hbm.25028 (PMC7416036; doi:10.1002/hbm.25028)
Supplement: Supplementary file 1 — Appendix S1. Supporting Information. [file HBM-41-3555-s001.pdf]

7 Supplementary Info

Algorithms	Sklearn Implementation
Feature Selection	
Principle Component Analysis	PCA
Fast algorithm for Independent Component Analysis	FastICA
Select the p-values corresponding to Family-wise error rate	SelectFwe
Select features according to a percentile of the highest scores	SelectPercentile
Remove low-variance Features	VarianceThreshold
Feature Generation	
Agglomerate features	FeatureAgglomeration
Regression	
Elastic Net model with iterative fitting along a regularisation path	ElasticNetCV
Randomised Decision Trees on sub-samples of the dataset	ExtraTreesRegressor
k-Nearest Neighbours Regression	KNeighborsRegressor
Cross-validated Lasso using the LARS algorithm	LassoLarsCV
Linear Support Vector Regression	LinearSVR
Linear Least squares with l2 regularisation	Ridge
Random Forest Regressor	RandomForestRegressor
Ordinary Least Squares Linear Regression	LinearRegression
Decision Tree Regressor	DecisionTreeRegressor
Gaussian process regression	GaussianProcessRegressors
Relevance Vector Regression	RVR$*$

Table S1: **List of used Feature Selection, Feature Generation and Regression Algorithms** ($*$: The RVR algorithm is not part of the Sklearn package but the used algorithm was provided by https://github.com/JamesRitchie/scikit-rvm)

Table S2: List of used Freesurfer features

lh bankssts thickness	lh caudalanteriorcingulate thickness	lh caudalmiddlefrontal thickness
lh cuneus thickness	lh entorhinal thickness	lh fusiform thickness
lh inferiorparietal thickness	lh inferiortemporal thickness	lh isthmuscingulate thickness
lh lateraloccipital thickness	lh lateralorbitofrontal thickness	lh lingual thickness
lh medialorbitofrontal thickness	lh middletemporal thickness	lh parahippocampal thickness
lh paracentral thickness	lh parsopercularis thickness	lh parsorbitalis thickness
lh parstriangularis thickness	lh pericalcarine thickness	lh postcentral thickness
lh posteriorcingulate thickness	lh precentral thickness	lh precuneus thickness
lh rostralanteriorcingulate thickness	lh rostralmiddlefrontal thickness	lh superiorfrontal thickness
lh superiorparietal thickness	lh superiortemporal thickness	lh supramarginal thickness
lh frontalpole thickness	lh temporalpole thickness	lh transversetemporal thickness
lh insula thickness	lh MeanThickness thickness	rh bankssts thickness
rh caudalanteriorcingulate thickness	rh caudalmiddlefrontal thickness	rh cuneus thickness
rh entorhinal thickness	rh fusiform thickness	rh inferiorparietal thickness
rh inferiortemporal thickness	rh isthmuscingulate thickness	rh lateraloccipital thickness
rh lateralorbitofrontal thickness	rh lingual thickness	rh medialorbitofrontal thickness
rh middletemporal thickness	rh parahippocampal thickness	rh paracentral thickness
rh parsopercularis thickness	rh parsorbitalis thickness	rh parstriangularis thickness
rh pericalcarine thickness	rh postcentral thickness	rh posteriorcingulate thickness
rh precentral thickness	rh precuneus thickness	rh rostralanteriorcingulate thickness
rh rostralmiddlefrontal thickness	rh superiorfrontal thickness	rh superiorparietal thickness
rh superiortemporal thickness	rh supramarginal thickness	rh frontalpole thickness
rh temporalpole thickness	rh transversetemporal thickness	rh insula thickness
Left-Cerebellum-White-Matter	Left-Cerebellum-Cortex	rh MeanThickness thickness
Left-Thalamus-Proper	Left-Caudate	Left-Putamen
Left-Pallidum	3rd-Ventricle	4th-Ventricle
Brain-Stem	Left-Hippocampus	Left-Amygdala
CSF	Left-Accumbens-area	Left-VentralDC
Left-vessel	Right-Cerebellum-White-Matter	Right-Cerebellum-Cortex
Right-Thalamus-Proper	Right-Caudate	Right-Putamen
Right-Pallidum	Right-Hippocampus	Right-Amygdala
Right-Accumbens-area	Right-VentralDC	Right-vessel
CC Posterior	CC Mid Posterior	CC Central
CC Mid Anterior	CC Anterior	rhCortexVol
CortexVol	lhCerebralWhiteMatterVol	rhCerebralWhiteMatterVol
CerebralWhiteMatterVol	SubCortGrayVol	TotalGrayVol
SupraTentorialVol	SupraTentorialVolNotVent	SupraTentorialVolNotVentVox
MaskVol	BrainSegVol-to-eTIV	MaskVol-to-eTIV
EstimatedTotalIntraCranialVol		

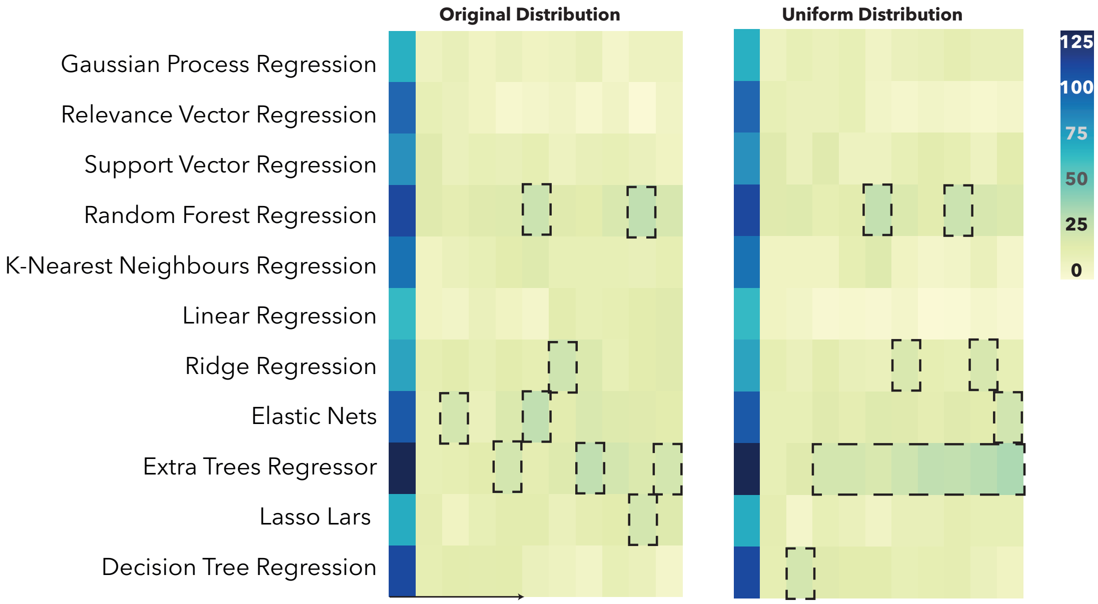

Figure S1: Model counts for the different distributions for one single repetition over the different generations. While **Left** shows the model count for the normal data distribution, **Right** illustrates the model counts for the uniform distribution. Using both distributions, the most common models explored by TPOT are Random Forest Regression, Extra Tree Regressors and Elastic Nets.
